# Severe Rhabdomyolysis Without Electrolyte Abnormalities or Renal Failure and With Disproportionately Low AST

**DOI:** 10.1155/carm/9601669

**Published:** 2026-07-16

**Authors:** Sarah Baig, Michelle Troendle

**Affiliations:** ^1^ Medical Student, Virginia Commonwealth University School of Medicine, Richmond, Virginia, USA, vcu.edu; ^2^ Department of Emergency Medicine, Virginia Commonwealth University Health, Richmond, Virginia, USA, vcu.edu

## Abstract

Rhabdomyolysis is a condition in which damaged muscle tissue breaks down and typically presents with muscle pain, weakness, and dark‐colored urine, along with markedly elevated serum creatine kinase (CK) levels. Common lab findings include hyperkalemia, hyperphosphatemia, hypocalcemia, elevated lactate dehydrogenase (LDH), acute kidney injury (AKI), and elevated aspartate aminotransferase (AST), with a typical CK:AST ratio of approximately 20:1. This report describes a 26‐year‐old man with no prior medical history who developed dark urine and lower back pain after a high‐intensity functional training (HIFT) session following a prolonged period of inactivity. Laboratory evaluation revealed an extraordinarily high CK of 260,100 U/L, AST of 2049 U/L, and ALT of 208 U/L, yielding a CK:AST ratio of approximately 127:1. Urinalysis showed myoglobinuria, while renal function and electrolytes remained normal throughout hospitalization. He was managed with intravenous fluids for 36 h, transitioned to oral hydration, and discharged on Day 6 with declining CK and AST levels. This case underscores that severe rhabdomyolysis may occur in the absence of renal dysfunction or electrolyte abnormalities and highlights the importance of prompt recognition and treatment even when traditional laboratory abnormalities are absent. The strikingly abnormal CK:AST ratio challenges reliance on expected lab patterns. Recognizing these atypical presentations is crucial for timely diagnosis and management. Early fluid resuscitation and close monitoring remain essential, even when routine findings appear normal.

## 1. Introduction

Rhabdomyolysis is a condition in which damaged muscle tissue breaks down, resulting in muscle pain, weakness, and swelling due to the release of skeletal muscle cell contents into the bloodstream [1, 2]. Diagnosis relies primarily through serum laboratory testing, with creatine kinase (CK) serving as the principal diagnostic marker [3]. Additional lab abnormalities include acute renal failure (ARF), elevated aspartate aminotransferase (AST) and alanine aminotransferase (ALT), hyperkalemia, hyperphosphatemia, hypocalcemia followed by hypercalcemia, and elevated lactate dehydrogenase (LDH) [1, 2, 4]. The CK:AST tends to increase proportionally due to its shared expression in skeletal muscle, with a typical ratio approximating 20:1 [5].

Common causes of rhabdomyolysis include trauma, toxins, viral infections, medications, extreme temperature changes, hypoxia, metabolic disturbances, and electrolyte imbalances [3]. Another recognized etiology is exercise‐induced rhabdomyolysis (EIR), which arises in individuals engaging in high‐intensity functional training (HIFT) or performing strenuous, unaccustomed exercise [1, 2, 4]. EIR following HIFT usually results in a less severe form of rhabdomyolysis with mild to moderate elevations in CK and AST [1, 6].

We report a case of EIR following HIFT with extremely elevated CK levels, a disproportionate CK:AST ratio, and no electrolyte abnormalities or renal impairment.

## 2. Case

A 26‐year‐old man with no known chronic medical history presented to the emergency department with dark‐colored urine and lower back pain following a session of HIFT performed with inadequate hydration. The exercise occurred after a prolonged period of physical inactivity due to recent ophthalmologic surgery. On examination, the patient appeared in no acute distress and had normal vital signs. Laboratory studies revealed a peak CK level of 260,100 U/L and AST of 2049 U/L, resulting in a CK:AST ratio of approximately 127:1. ALT was also elevated at 208 U/L. Urinalysis was positive for heme without red blood cells, consistent with myoglobinuria. Renal function and electrolytes were within normal limits. The patient had no personal or family history of myopathy, prior episodes of rhabdomyolysis, endocrine disorders, substance use, supplement use, or medication exposures known to precipitate rhabdomyolysis. Thyroid studies obtained during hospitalization were within normal limits (TSH 2.11 mIU/L and free T4 0.8 ng/dL). Given the clear temporal relationship between a HIFT session following a prolonged period of inactivity after recent ophthalmologic surgery and the onset of symptoms, EIR was considered the most likely etiology.

**FIGURE 1:**
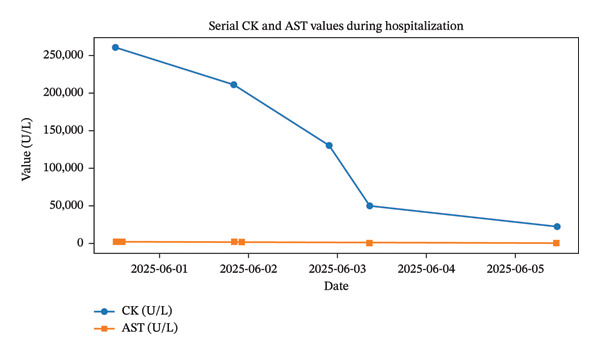
Trends in serum creatine kinase (CK) and aspartate aminotransferase (AST) levels during hospitalization. Both biomarkers demonstrated a progressive decline throughout hospitalization following intravenous fluid therapy.

The patient was treated with continuous intravenous fluid therapy for 36 h, followed by a transition to oral hydration. He remained free of electrolyte disturbances or renal dysfunction throughout his hospital stay. By Day 6, CK had decreased to 22,920 U/L, and AST had declined to 390 U/L. He was discharged on hospital Day 6 in stable condition. Serial CK and AST trends during hospitalization are shown in Figure 1.

## 3. Discussion

This case presents several distinctive features. First, while EIR is typically considered a milder form of the condition, this patient exhibited an extremely elevated CK level. Second, despite the severity of muscle injury, there were no signs of renal failure or electrolyte abnormalities, which are usually expected in such presentations. Third, the patient demonstrated an unusually high CK:AST ratio, deviating from conventional patterns. The absence of expected biochemical derangements supports the possibility of an atypical pathophysiologic variant of rhabdomyolysis. Although inherited metabolic myopathies and endocrine disorders may predispose patients to exertional rhabdomyolysis, the absence of recurrent episodes, family history, or other clinical indicators made these diagnoses less likely in this patient. Follow‐up laboratory values after discharge were unavailable; therefore, the exact timeline to complete CK normalization could not be determined.

The most probable cause of this patient’s severe rhabdomyolysis was intense physical exertion without adequate hydration during his HIFT, following a prolonged period of immobility. His preserved renal function could be attributed to partial, but not adequate, self‐rehydration postexercise or his status as a young, otherwise healthy individual capable of physiologic compensation. A plausible explanation for the absence of electrolyte abnormalities involves selective muscle cell injury, where cytosolic enzymes were released into the circulation while the sarcolemma remained largely intact. This preservation of membrane integrity would have restricted the efflux of ionic constituents and other intracellular solutes [5, 7, 8]. The markedly elevated CK:AST ratio (∼127:1) supports this mechanism, indicating enzyme leakage in the absence of widespread membrane disruption.

Although this case does not reflect the highest reported CK level, the value was nonetheless profoundly elevated, highlighting the potential severity of EIR. Importantly, the presence of normal serum electrolytes and preserved renal function does not exclude significant pathology. This case also reinforces the importance of early recognition and prompt fluid resuscitation. Despite an initial CK level exceeding 260,000 U/L, the patient maintained normal renal function and electrolyte values throughout hospitalization. While causality cannot be established from a single case, the favorable clinical course may have been influenced by timely supportive management with intravenous fluids. Clinicians should maintain a high index of suspicion for rhabdomyolysis in postexercise patients, even when adjunctive laboratory markers appear within normal limits. Early recognition, prompt initiation of fluid therapy, and confirmatory CK testing remain essential to prevent delayed diagnosis and reduce the risk of complications.

## Funding

No funding was received for this manuscript.

## Conflicts of Interest

The authors declare no conflicts of interest.

## Data Availability

The data that support the findings of this study are available on request from the corresponding author. The data are not publicly available due to privacy or ethical restrictions.
